# Validation of the new ABC score for predicting 30-day mortality in gastrointestinal bleeding

**DOI:** 10.1186/s12876-022-02374-y

**Published:** 2022-06-21

**Authors:** Marie Christelle Saade, Anthony Kerbage, Suha Jabak, Maha Makki, Kassem Barada, Yasser Shaib

**Affiliations:** 1 Division of Gastroenterology and Hepatology, Department of Internal Medicine, American University of Beirut Medical Center, Beirut, Lebanon; 2 Biostatistics Support Unit, Clinical Research Institute, American University of Beirut Medical Center, Beirut, Lebanon

**Keywords:** Gastrointestinal bleeding, ABC score validation, Gastrointestinal bleeding outcomes

## Abstract

**Background/Aim:**

The ABC score is a new pre-endoscopic scoring system that was recently developed to accurately predict one-month mortality in upper and lower gastrointestinal bleeding (GIB). We aim to validate this new score on a cohort of Lebanese patients treated in a tertiary care center and to compare it to currently existing scores.

**Methods:**

Adult patients admitted to the American University of Beirut Medical Center (AUBMC) with overt GIB between January 2013 and August 2020 were included. The area under receiver operating characteristic (AUROC) curves of the ABC score in predicting 30-day mortality was calculated using the SPSS software. Other optimal existing scores for predicting mortality (the Oakland score for lower GIB, the AIMS-65 and the Rockall scores for upper GIB)s were also assessed and compared to the ABC score.

**Results:**

A total of 310 patients were included in our study. For upper GIB, the ABC score showed good performance in predicting 30-day mortality (AUROC: 0.79), outperforming both the AIMS-65 score (AUROC 0.67, *p* < 0.001) and the Rockall score (AUROC: 0.62, *p* < 0.001). For lower GIB, the ABC score also had good performance which was comparable to the Oakland score (AUROC: 0.70 vs 0.56, *p* = 0.26).

**Conclusion:**

In our cohort of patients, the ABC score demonstrated good performance in predicting 30-day mortality for patients with upper and lower GIB compared to other established risk scores, which may help guide management decisions. This simple and novel score provides valuable prognostic information for patients presenting with GIB and appears to be reproducible in different patient populations.

## Introduction

Acute gastrointestinal bleeding (GIB) is a frequently encountered medical emergency with significant morbidity and mortality [1]. The physician in charge is challenged to make a rapid diagnosis, a thoughtful risk assessment and an efficient resuscitation in order to improve outcomes and limit the risk of complications [2].

Multiple prognostic scoring systems have been developed to identify high-risk patients and low-risk patients presenting with GIB and are widely used in emergency departments for triaging patients. Identifying low-risk patients who can be managed electively or on an outpatient basis decrease the burden on physicians, patients and the health care system [3]. On the other hand, recognizing high-risk patients who require immediate hospitalization and intervention helps prevent delays in management, thus decreasing morbidity and mortality [4].

The AIMS65 and Progetto Nazionale Emorragia Digestiva (PNED) have been shown to most accurately predict mortality among patients with upper gastrointestinal bleeding (UGIB) [5]. The Glasgow-Blatchford score is superior to other scores in identifying low-risk patients who could be managed on an outpatient basis [6]. Overall, prognostic scores have a higher accuracy in non-variceal UGIB compared to variceal bleeding [7]. In lower gastrointestinal bleeding (LGIB), most prognostic scores are poor at predicting mortality with modest area under receiver operating characteristic (AUROC) curves [8]. According to the British Society of Gastroenterology, the newly developed Oakland score is the preferred scoring system for predicting mortality in LGIB [9].

It is often challenging to accurately confirm the location of GIB (upper or lower GIB) upon presentation before endoscopic investigation, which makes it difficult to use prognostic scores that are specific to bleeding location. Ideally one prognostic score applicable in upper and lower GIB irrespective of the etiology would be most useful clinically [10]. The recent UK National Confidential Enquiry into Patient Outcome and Death (NCEPOD) recommends the development of one score that could help direct the management of any patient presenting with GIB independently of the bleeding location.

In 2020, Laursen SB et al. developed the Age, Blood tests and Comorbidities (ABC) score, which was appealing in that it is relatively simple to calculate, and accurately predicts 30-day mortality in patients presenting with both upper and lower GIB. The ABC was also shown to have a superior performance when compared to previously established prognostic scores [10]. Physicians managing acute GIB are still unaccustomed with the ABC score because it is still relatively new, and was developed during the COVID-19 pandemic [11]. Additionally, few studies have attempted to validate it on different patient populations.

Thus, in this study, we aimed to validate the ABC score in patients presenting with GIB to the American University of Beirut Medical Center (AUBMC). Our goal is to define its accuracy in risk-stratification and prediction of mortality compared to previously developed prognostic scores.

## Methods

### Study design and population

This is a single center cohort study that included all adult patients admitted to AUBMC with overt GIB between January 2013 and August 2020. The data was obtained from a database that prospectively includes patients presenting with GIB to our center.

Patients younger than 18 years old, patients with known Inflammatory Bowel Disease (IBD), pregnant women, and/or patients presenting with occult GIB were excluded. The definition of overt upper or lower GIB included hematemesis, coffee ground emesis, melena, and hematochezia. Bleeding was considered upper gastrointestinal bleeding (UGIB) when coffee ground emesis or hematemesis was reported and/or stigmata of recent hemorrhage (SRH) was shown in the upper gastrointestinal tract by endoscopy.

Bleeding was considered lower gastrointestinal bleeding (LGIB) when hematochezia was reported and/or SRH was demonstrated in the colon by colonoscopy with no other UGIB source.

Data collected included demographics (age, gender), comorbidities (ischemic heart disease, diabetes, liver cirrhosis, renal failure, malignancy), age-adjusted Charlson Comorbidity Index (CCI) score, home medications (including aspirin, non-aspirin anti-platelets, anticoagulants or non-steroidal anti-inflammatory drugs), vital signs upon presentation, physical examination on presentation (level of consciousness, abdominal examination, digital rectal examination), symptoms occurring within 72 h and 30 days of presentation, initial laboratory data (hemoglobin, urea, creatinine, albumin), blood transfusion, findings of endoscopy, location of GIB and all-cause death within 30 days. After one month duration, the follow up was performed by calling patients on their personal number. If patients were already deceased or did not respond after multiple trials, we used to address their emergency contact.

In order to calculate the ABC score we substituted the value of CCI to its equivalent in the ASA score based on the categorization of severity. A CCI score ≤ 2 is considered mild in severity and was considered equivalent to an ASA score of 1–2. Similarly, a CCI score of 3 or 4 (moderate) was substituted by an ASA score of 3 and a CCI score ≥ 5 (severe) by an ASA score of 4. [12, 13]

### Statistical analysis

The Statistical Package for the Social Sciences (SPSS) version 23.0 was used for data cleaning, management and analysis. Descriptive statistics are expressed as means, and standard deviation (± SD) for continuous variables and frequencies and percentages for categorical variables. The comparison of different AUROCs and generation of p-values and figures was done using Stata Statistical Software.

#### Validation of the ABC risk score in UGIB and LGIB

External validation of the ABC score’s ability to predict 30-day mortality in upper and lower GIB was performed by evaluating the AUROC (area under receiver operating characteristic), sensitivities and specificities using the above-described cohort.

#### Comparison of the performance of the ABC risk score with optimal existing scores for predicting mortality

The discriminative ability of the ABC score in predicting 30-day mortality in upper GI bleed was compared with both the ROCKALL and the AIMS-65 scores using the AUROCs. In prediction of the mortality in lower GIB, the ABC score was compared with the OAKLAND score using the AUROCs as well.

## Results

### Patients characteristics

A total of 310 patients were included, 199 (63%) were men and 111 (37%) were women with a mean age of 68.16 ± 17.1 years. The mean age-adjusted CCI for all included patients was 2.6. Of all patients, 198 (63.9%) received blood transfusion, 88 (28.4%) were on antiplatelet upon admission, 62 (20%) on anticoagulation and 41 (13.2%) on both antiplatelet and anticoagulation medications. Among all included patients, 185 (59.7%) had UGIB, 99 (31.9%) had LGIB and 26 (8.4%) had GIB of unspecified location. After a one-month period, 22 deaths (16.3%) were recorded among upper GIB patients, and 10 deaths were recorded among lower GIB patients (10.1%), with cardiovascular death and sepsis being the most frequent causes of death in both groups. Moreover 6 deaths were noted with unknown GI bleeding location.

The detailed characteristics of all included patients are shown in Table 1.

**Table 1:** Baseline demographic and clinical characteristics and outcomes of patients presenting with gastrointestinal bleeding

Female gender – no. (%)	111 (35.8)
Mean age – yr. (sd)	68.16 (17.1)
Mean age-adjusted CCI – (sd)	2.6 (2.3)
AT upon presentation – no. (%)	
AP only	88 (28.4)
AC only	62 (20.0)
AP and AC	41 (13.2)
NSAIDs – no. (%)	29 (9.4)
GIB Location – no. (%)	
UGIB	185 (59.7)
LGIB	99 (31.9)
Mean initial SBP – mmHg (sd)	123.3 (22.0)
Mean initial hemoglobin – g/dL (sd)	9.4 (2.7)
Mean BUN – mg/dL (sd)	40.5 (30.2)
Mean creatinine – mg/dL (sd)	1.5 (1.5)
Mean albumin – g/L (sd)	32.4 (6.9)
Transfusion – no. (%)	198 (63.9)
1-month mortality – no. (%)	38 (12.3)
Among UGIB	22 (16.3)
Among LGIB	10 (10.1)
Causes of mortality – no. (%)	
Among UGIB	
Cardiovascular	5 (22.7)
Sepsis	9 (40.9)
Gastrointestinal bleeding	2 (9.1)
Systemic cancer	4 (18.2)
Multiorgan failure	2 (9.1)
Among LGIB	
Cardiovascular	1 (11.1)
Sepsis	4 (44.4)
Gastrointestinal bleeding	1 (11.1)
Systemic cancer	1 (11.1)
Multiorgan failure	2 (22.2)

*Age-Adjusted CCI*, *Age-adjusted Charlson Comorbidity Index, AT, Antithrombotic, AP, Antiplatelet, AC, Anticoagulant, NSAIDs, Non-steroidal anti-inflammatory drugs, GIB, Gastrointestinal bleeding, UGIB, Upper gastrointestinal bleeding, LGIB, Lower gastrointestinal bleeding, SBP, Systolic blood pressure, Hb, Hemoglobin*

The causes of gastrointestinal bleeding were diverse. The most frequent causes among patients with UGIB were peptic ulcer disease and esophageal/gastric varices with 90 (48.6%) and 31 (16.8%) patients respectively. As for the group with LGIB, diverticulosis and hemorrhoids were the most reported with 25 (25.3%) and 12 (12.1%) patients respectively.

The causes of bleeding for UGIB and LGIB patients are detailed in Table 2 and 3.

**Table 2:** Causes of bleeding in UGIB patients

Cause of bleeding in UGIB patients – no. (%)	UGIB
Peptic ulcer disease	90 (48.6)
Esophageal/gastric varices	31 (16.8)
Unknown	28 (15.1)
Luminal GI cancer	11 (5.9)
Arterio-venous malformation	11 (5.9)
Dieulafoy’s lesion	5 (2.7)
Mallory Weiss tear	2 (1.1)
Mesenteric ischemia	2 (1.1)
Anastomotic lesions	2 (1.1)
Cameron lesion	1 (0.5)
Polyp	1 (0.5)
Post-sphincterotomy	1 (0.5)

**Table 3:** Causes of bleeding in LGIB patients

Cause of bleeding in LGIB patients – no. (%)	UGIB
Diverticulosis	25 (25.3)
Hemorrhoids	12 (12.1)
Unknown	12 (12.1)
Luminal GI cancer	11 (11.1)
Arterio-venous malformation	10 (10.1)
Colitis	6 (6.1)
Polyp	6 (6.1)
Peptic ulcer disease	6 (6.1)
Post-polypectomy	2 (2)
Colonic ulcer	2 (2)
Dieulafoy’s lesion	2 (2)
Ischemic colitis	1 (1)
Meckel's diverticulum	1 (1)
Post-sphincterotomy	1 (1)
Radiation proctitis	1 (1)
Anal fissure	1 (1)

### Score performance

The ABC score is classified as low (≤ 3), medium (4–7) and high risk (≥ 8).(11) In fact, 86, 176 and 48 of our patients were respectively characterized as low, medium and high risk for 30-day mortality as to their ABC score. The proportion of death corresponding to low (2.3%), medium (13.1%) and high (27.1%) risk patients was shown significantly different between the three groups (p-value < 0.001) as well as in harmony with the severity of the risk assessment. These results are detailed in Table 4.

**Table 4:** ABC Score compared to the relative risk of mortality. (*p* < 0.001)

ABC Score 30-day mortality risk	Number of total patients (%)	Number of 30-days mortality patients (%)
Low risk (≤ 3)	86 (27.7%)	2 (2.3%)
Medium risk (4–7)	176 (56.7%)	23 (13.1%)
High risk (≥ 8)	48 (15.4%)	13 (27.1%)

#### Upper GI bleeding

Among patients with UGIB, the ABC score **(AUROC 0.79)** showed the best discriminative ability for predicting 30-day mortality compared to the AIMS-65 score (AUROC 0.67; *p*-value < 0.001) and to the Rockall score (AUROC 0.62; *p*-value < 0.001).

#### Lower GI bleeding

Among patients with LGIB, the ABC score **(AUROC 0.7)** demonstrated a satisfactory performance in predicting 30-day mortality and was comparable to the Oakland score (AUROC 0.56; *p*-value = 0.26).

**Fig. 1:**
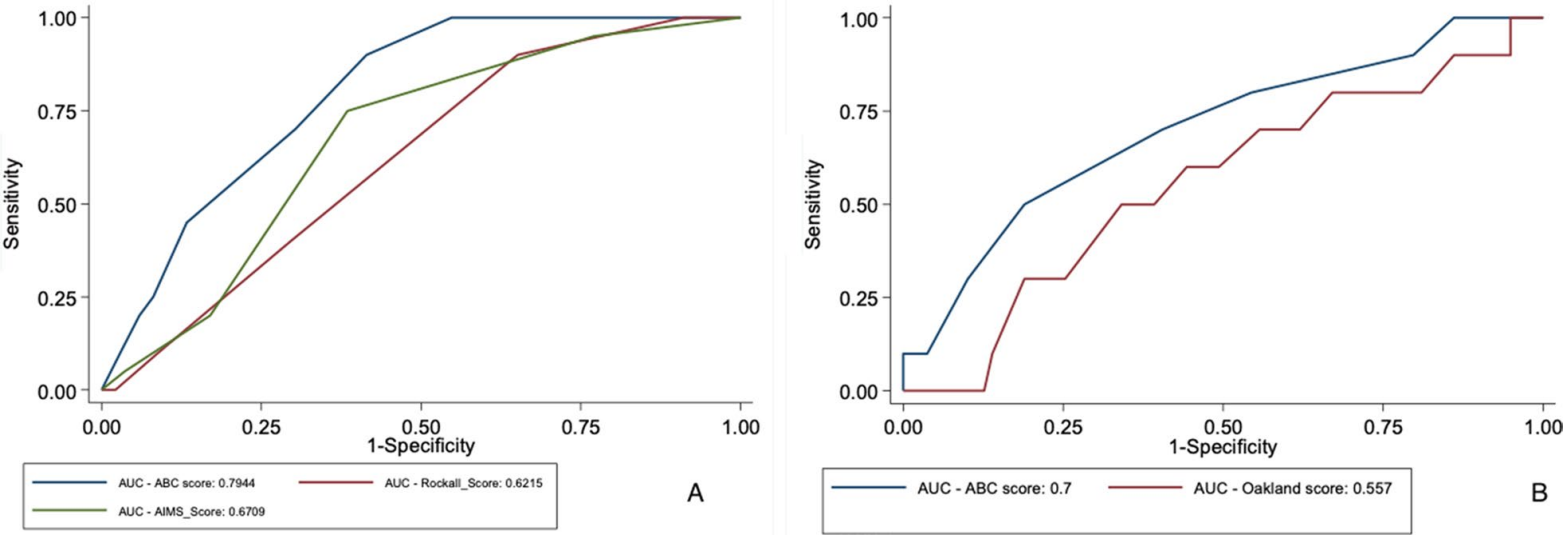
Comparison of ABC score with different scores. **A**. Comparison of ABC, AIMS65 and Rockall scores in prediction of 30-day mortality in upper gastrointestinal bleeding. **B**. Comparison of ABC and Oakland scores in prediction of 30-day mortality in lower gastrointestinal bleeding

Figure 1 shows the performance of the ABC score in upper and lower GI bleeding patients.

The means of the prognostic scores for upper and lower GI bleeding (AIMS-65, Rockall, GBS, ABC and Oakland score) are disclosed in Table 5.

**Table 5:** Prognostic scores of UGIB and LGIB patients

	Mean in UGIB patients (S.D.)	Mean in LGIB patients (S.D.)
AIMS-65 score	1.44 (1.08)	1.55 (1.05)
Rockall score	3.84 (1.12)	–
GBS score	9.53 (3.93)	6.81 (4.50)
ABC score	5.26 (2.60)	4.94 (2.28)
Oakland score	–	19.24 (6.44)

*S.D. Standard Deviation*

## Discussion

In 2020, the ABC score was developed to accurately predict 30-day mortality in patients presenting with GIB. Compared to previously used scores, the ABC score can be calculated early after patient presentation and has a good performance in predicting mortality in patients with both UGIB and LGIB.

This recently developed score needs to be validated on various populations in different settings to prove its superiority. In our study, we analyzed the performance of this score in the Lebanese population among a cohort of patients presenting with GIB and compared it to other existing scores.

In our cohort, the ABC score demonstrated the best performance in predicting 30-day mortality for patients with UGIB when compared to other calculated scores. Our results came in harmony with previous studies. In fact, according to Laursen et al. the ABC score in UGIB patients (AUROC 0.81) performed better than any of the available UGIB scores for predicting 30-day mortality [10]. Moreover, in three additional studies by Mules et al., Saffouri et al. and Liu et al., the ABC score outperformed all other scores for 30-day mortality in patients with UGIB with an AUROC of 0.85, 0.86 and 0.72 respectively [14–16].

For LGIB, few studies have investigated the performance of ABC score in predicting mortality. According to Laursen et al., the ABC score is superior to the existing LGIB scores. In our population the ABC score was shown comparable to the Oakland score (AUROC 0.7).

Based on our findings, we endorse the use of the ABC score in lieu of traditional scores to stratify the severity of the presentation.

There are some limitations to our study. First, this was a single center study, and the number of patients was small. We initially had a larger cohort of patients presenting with GIB, but we could not include them all because of missing albumin and INR levels. This may have introduced some unwanted bias and has reduced our sample size. In addition, most of our patients presented with UGIB leading to a smaller number of patients with LGIB. This may explain why the difference in performance between the ABC and Oakland scores in LGIB did not reach statistical significance. For this reason, further cohorts with larger number of patients are needed to validate the use of this new score in LGIB patients. Second, since the ASA score, one of the components of the ABC score, was not determined for all patients upon admission, we instead used the age-adjusted Charlson Comorbidity Index (CCI) as an equivalent [12]. Although these scores have some differences [17], Lavelle et al. report a excellent agreement between them [13].

So far, the ABC score seems superior to all other existing scores for predicting 30-day mortality in UGIB and has a good performance in predicting 30-day mortality in LGIB. This tool is useful to help physicians identify high-risk patients and adapt an aggressive management plan and close monitoring.

## Data Availability

The datasets used and/or analyzed during the current study will be available from the corresponding author on reasonable request.
